# Supplementary material for: Associations between Dynamic Vitamin D Level and Thyroid Function during Pregnancy
Source: Nutrients. 2022 Sep 14;14(18):3780. doi: 10.3390/nu14183780 (PMC9504818; doi:10.3390/nu14183780)
Supplement: Supplementary file 1 [file nutrients-14-03780-s001.zip › nutrients-1908473-supplementary.pdf]

Table S1. Thyroid function in the quartile of VitD level [1] quartile in each trimester.

Indicators	Q1 (2.4,13.1)	Q2 (13.1,18.3)	Q3 (18.3,24.3)	Q4 (24.3,92.9)	*P*-value[2]
**First trimester(n=8828)**	**n=2224**	**n=2196**	**n=2226**	**n=2182**	
VitD level	10.30 (3.30)[3]	15.80 (2.70)	21.30 (3.00)	28.30 (5.40)	<0.001
TPOAb(IU/mL)	0.32 (0.57)	0.33 (0.57)	0.36 (0.61)	0.37 (0.70)	0.964
TSH (μIU/ml)	0.87 (0.97)	0.91 (0.97)	0.92 (0.93)	0.93 (0.93)	0.107
fT3 (pmol/L)	4.17 (0.64)	4.19 (0.60)	4.24 (0.62)	4.28 (0.64)	<0.001
fT4 (pmol/L)	13.10 (2.09)	13.29 (2.09)	13.27 (1.97)	13.44 (2.06)	0.094
**Second trimester (n=1396)**	**n=379**	**n=327**	**n=328**	**n=362**	
TSH (μIU/ml)	0.91 (0.83)	0.85 (0.81)	0.84 (0.81)	0.84 (0.84)	0.585
fT3 (pmol/L)	4.08 (0.64)	4.08 (0.60)	4.10 (0.62)	4.04 (0.54)	0.437
fT4 (pmol/L)	11.21 (1.74)	11.22 (1.80)	11.12 (1.71)	11.29 (1.79)	0.226
**Third trimester (n=562)**	**n=129**	**n=132**	**n=160**	**n=141**	
TSH (μIU/ml)	1.31 (1.14)	1.18 (1.03)	1.32 (1.19)	1.28 (1.16)	0.200
fT3 (pmol/L)	3.89 (0.63)	3.74 (0.63)	3.84 (0.62)	3.77 (0.59)	0.138
fT4 (pmol/L)	10.06 (1.89)	9.92 (1.67)	9.85 (1.97)	9.91 (1.58)	0.769

[1]The VitD level is based on the measurement of the first trimester.

[2] Continuous variables were compared by using One-way ANOVA test for normal distribution data or Kruskal-Wallis rank sum test for skew distribution data among the 4 subgroups.

[3]Values are present as median (IQR).

Table S2. Comparison of characteristics between the population of the total population and the GEE population

Characteristic	N = 8,616	N = 212	P^2
Maternal age	29.40 (5.00)[1]	29.60 (5.17)	0.477
Maternal education			0.014
Low(high school or below)	1,291 (15.0)	17 (8.0)	
Middle (college)	3,734 (43.3)	94 (44.3)	
High (university or above)	3,591 (41.7)	101 (47.6)	
Maternal employment			0.323
Unemployed	952 (11.0)	28 (13.2)	
Employed	7,664 (89.0)	184 (86.8)	
Race/ethnicity			0.897
Han	8,106 (94.1)	199 (93.9)	
Other	510 (5.9)	13 (6.1)	
Parity			0.171
Primiparous	4,918 (57.1)	131 (61.8)	
Multiparous	3,698 (42.9)	81 (38.2)	
Maternal BMI class			0.685
Underweight	819 (9.5)	21 (9.9)	
Normal	5,677 (65.9)	146 (68.9)	
Overweight	1,679 (19.5)	37 (17.5)	
Obesity	441 (5.1)	8 (3.8)	
Folate supplement 3-months before and after conception			0.121
No	748 (8.7)	12 (5.7)	
Yes	7,868 (91.3)	200 (94.3)	
Season of conception			0.124
Spring	1,926 (22.4)	48 (22.6)	
Summer	1,584 (18.4)	47 (22.2)	
Autumn	2,545 (29.5)	69 (32.5)	
Winter	2,561 (29.7)	48 (22.6)	
Vitamin D (0-13 weeks), ng/ml	18.30 (11.10)	19.85 (11.55)	0.089
Vitamin D deficiency (0-13 weeks)			0.047
No	3,678 (42.7)	105 (49.5)	
Yes	4,938 (57.3)	107 (50.5)	
Vitamin D (14-28 weeks), ng/ml	30.50 (16.30)	30.80 (14.73)	0.266
Vitamin D deficiency (14-28 weeks)			0.037
No	6,899 (80.1)	182 (85.8)	
Yes	1,717 (19.9)	30 (14.2)	
Vitamin D supplement during pregnancy			0.555
No	793 (9.2)	17 (8.0)	
Yes	7,823 (90.8)	195 (92.0)	
Multivitamin supplement during pregnancy			0.125
No	4,198 (48.7)	92 (43.4)	
Yes	4,418 (51.3)	120 (56.6)	
Gestational Hypertensive Disorder	176 (2.0)	9 (4.2)	0.045
Gestational Diabetes Mellitus	2,412 (28.0)	80 (37.7)	0.002
**First trimester(n=8616)**			
TPOAb (IU/mL)	0.35 (0.60)	0.56 (62.64)	<0.001
TPOAb positive diagnosis			<0.001
No	7,690 (89.3)	145 (68.4)	
Yes	926 (10.7)	67 (31.6)	

Characteristic	N = 8,616	N = 212	*P*[2]
TSH (μIU/ml)	0.92 (0.94)	0.23 (0.95)	<0.001
fT3 (pmol/L)	4.21 (0.62)	4.53 (1.01)	<0.001
fT4 (pmol/L)	13.27 (2.04)	14.34 (3.55)	<0.001
**Second trimester(n=1184)**			
TSH (μIU/ml)	0.87 (0.80)	0.83 (1.13)	0.157
fT3 (pmol/L)	4.07 (0.61)	4.02 (0.65)	0.491
fT4 (pmol/L)	11.22 (1.73)	11.20 (2.13)	0.659
**Third trimester (n=350)**			
TSH (μIU/ml)	1.42 (1.17)	1.04 (0.97)	<0.001
fT3 (pmol/L)	3.80 (0.60)	3.88 (0.71)	0.093
fT4 (pmol/L)	9.74 (1.66)	10.20 (1.70)	<0.001

[1]Values are n (%) for categorical variables and median (IQR) for a continuous variable with a skewed distribution. Vitamin D concentration was measured with 25-hydroxyvitamin D in the serum.

[2] Continuous variables were compared by using One-way ANOVA test for normal distribution data or Kruskal-Wallis rank sum test for skew distribution data among the 4 subgroups.

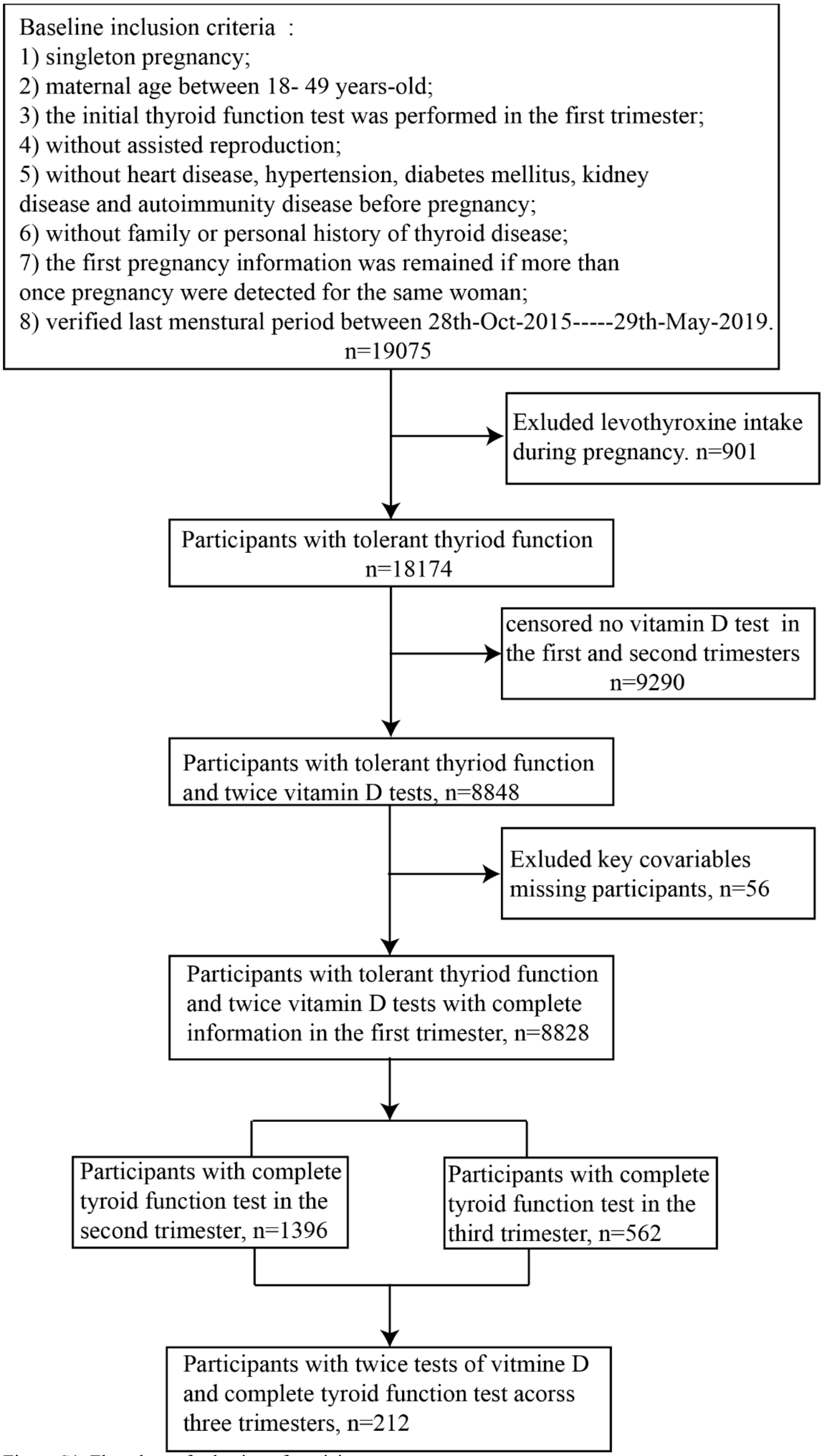

Figure S1. Flowchart of selection of participants.

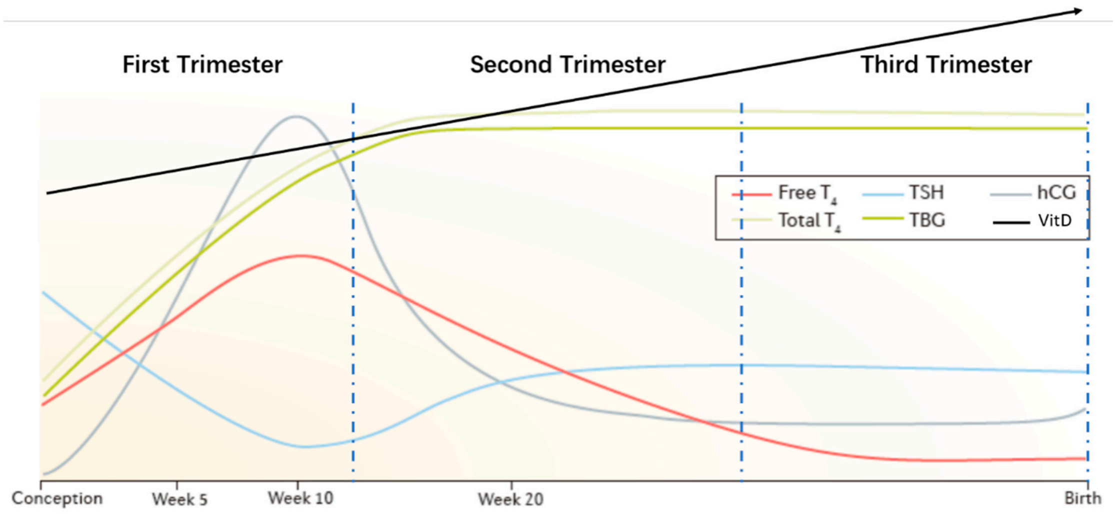

Figure S2. Schematic diagram of theoretically physiological changes of vitamin D and thyroid parameters. (Modified from thyroid disease in pregnancy: new insights in diagnosis and clinical management.)
